# A Process for the Design and Development of Novel Bone Morphogenetic Protein-7 (BMP-7) Mimetics With an Example: THR-184

**DOI:** 10.3389/fphar.2022.864509

**Published:** 2022-07-08

**Authors:** William D. Carlson, Peter C. Keck, Dattatreyamurty Bosukonda, Frederic Roy Carlson

**Affiliations:** ^1^ Division of Cardiology, Mass General Hospital/Harvard, Boston, MA, United States; ^2^ Harvard Medical School, Boston, MA, United States; ^3^ Therapeutics By Design, Boston, MA, United States; ^4^ Thrasos Therapeutics, Hopkinton, MA, United States

**Keywords:** Bone Morphogenetic Protein: BMP, Transforming Growth Factor: TGF, Cardio-Vascular Surgery: CVS, Acute Kidney Injury: AKI, Bone Morphogenetic Protein Mimetics: BMP Mimetics, THR-123, THR-184, Epithelial to Mesenchymal Transformation: EMT

## Abstract

Growth Factors have been evaluated as therapeutic targets for the treatment of a broad spectrum of diseases. Because they are proteins with pleiotropic effects, the quest to harness their beneficial effects has presented challenges. Most Growth Factors operate at the extracellular-receptor level and have natural feedback mechanisms that modulate their effects. As proteins, they are difficult and expensive to manufacture. Frequently proteins must be administered parenterally, may invoke an immune response, and may be neutralized by naturally occurring inhibitors. To circumvent these limitations, we have undertaken an effort to develop mimetics for the Bone Morphogenetic Protein (BMP) signaling pathway effects that incorporate the beneficial effects, eliminate the deleterious effects, and thereby create effective drug-like compounds.To this end, we have designed and tested a family of small peptide BMP mimetics. The design used the three-dimensional structure of BMP-7 to identify likely active surface regions. Lead sequences were then optimized based on *in vitro* assays that examine the selective binding to BMP receptors, demonstrate the phosphorylation of Smad-1,5,8, detect anti-apoptosis and anti-inflammation, and block the epithelial to mesenchymal transition (EMT) in renal tubular epithelial cells. These sequences were further optimized using *in vivo* assays of the attenuation of acute kidney injury in a rat-model of unilateral clamp ischemic reperfusion. This process uses a Structure Variance Analysis algorithm (SVA) to identify structure/activity relationships. One member of this family, THR-184, is an agonist of BMP signaling and a potent antagonist of TGFβ signaling. This small peptide mimetic inhibits inflammation, apoptosis, fibrosis and reverses epithelial to mesenchymal transition (EMT) by regulating multiple signaling pathways involved in the cellular injury of multiple organs. Its effects have been shown to control Acute Kidney Injury (AKI). THR-184 has progressed through phase I and II clinical trials for the prevention of Cardio-Vascular Surgery (CVS) associated AKI. This work provides a roadmap for the development of other growth factor mimetics and demonstrates how we might harness their therapeutic potential.

## 1 Introduction

Homeostasis, both during development and in the adult, is achieved through a careful balance of signaling pathways which transmit signals through ligands circulating through the body and then through cell surface receptors and their downstream signaling pathways. Most human diseases arise from either inappropriate activation or inhibition of these signaling pathways. One of the ubiquitous regulators of physiological, and cellular processes is the transforming growth factor β (TGF-β) superfamily. The TGF-β superfamily contains more than 30 structurally related polypeptide growth factors including TGF-βs, bone morphogenetic proteins (BMPs), activins (A, B), inhibins (A, B), and growth differentiation factors including myostatin and nodal (Leask and Abraham, 2004; Massague et al., 2005; Morty et al., 2009; Massague, 2012). The TGF-β superfamily normally functions to regulate embryonic development and cellular homeostasis, including regulation of proliferation, differentiation, apoptosis, and extracellular matrix remodeling in a cell specific manner (Siegel and Massague, 2003; de Caestecker, 2004; Massague and Gomis, 2006; Peng et al., 2022). Some of the most difficult disease conditions to treat are multifactored and involve tissue repair and/or differentiation. An example is the reperfusion injury following an ischemic event. In such an event, antagonists have often been used to regulate a single signaling pathway involved (Neely and Keith, 1995; Schulz et al., 2001; Pachori et al., 2010), but with limited success. A better therapeutic approach would be to regulate the effects of the multiple pathways involved by modulating them naturally through the activation of selective cell surface BMP receptors.

Our hypothesis is that we can develop small peptide mimetics of BMP that are effective against multiple diseases, for example, Acute Kidney Injury (AKI), LV dysfunction in aortic stenosis, Diabetes, Diabetic nephropathy, Idiopathic Pulmonary Fibrosis (IPF) and Pulmonary Arterial Hypertension (PAH).

To test this hypothesis, we embarked on a project wherein we designed and developed peptide mimetics of Bone Morphogenetic Protein-7 (BMP-7) for the treatment of Acute Kidney Injury (AKI). There are several reasons for the choice of BMP-7. In general, repair and regeneration of a tissue reflect many aspects of its embryologic development. BMP-7 is an important developmental factor for soft tissues such as the kidney. The generation of BMP-7-deficient mice has provided vital evidence that this growth factor regulates many morphogenetic processes including, but not limited to, skeletal development. BMP-7 acts as an early inducer of glomeruli formation and is required for skeletal patterning. Not only is BMP-7 involved in the differentiation of several organs during development but also suggests that mutations in the BMP seven gene itself, or in the genetic pathway, could be responsible for several human genetic diseases in which glomerulus formation is impaired (Luo et al., 1995). BMP-7 and TGF-beta act through specific receptors inducing distinct SMAD signaling pathways which are responsible for maintaining homeostasis of epithelial and endothelial tissues. The dysfunctional regulation of BMP and TGF-β signaling contributes to developmental anomalies and multiple diseases (Cao et al., 2019). The BMP and TGF-β signaling pathways often oppose one another’s effects in disease states (Hudnall et al., 2016). For example, BMP-7 reduces the TGF-β2-induced accumulation of fibrillar extra-cellular matrix (ECM) compounds in the human trabecular meshwork by antagonizing the fibrogenic effects of TGF-β signaling (Fuchshofer et al., 2007).

Here, we have taken a novel approach to the design of BMP-7 mimetics. In this approach, peptide mimetics are designed to target the control of various pathological processes such as inflammation, apoptosis, EMT and fibrosis that are critically involved in acute and chronic diseases. Also, considered in the design is, the elimination of the bone-inducing property of BMP which is an adverse event in soft tissues. Ischemia and reperfusion injury often results in AKI, a pernicious condition that when extensive enough, leads to the loss of renal function and increased mortality. Furthermore, if AKI remains untreated it can progress into chronic kidney disease (CKD) which can lead to a higher morbidity and mortality (Basile et al., 2012; Devarajan and Jefferies, 2016; Hsu and Hsu, 2016). A set of studies (Cutroneo, 2007; Piersma et al., 2015) has shown that the BMP pathway opposes TGF-β–induced fibrosis and promotes tissue recovery in several of these clinically relevant fibrotic diseases and CKD.

THR-184 is an optimized BMP-7 mimetic that we chose to test for safety and efficacy in pre-clinical and clinical Phase I and II trials. The phase II clinical trial (NCT01830920) evaluated THR-184 for its safety and efficacy in preventing AKI in ‘at risk’ patients undergoing cardio-vascular surgery that employs a cardio-pulmonary bypass pump (CPB). The recruitment of these patients was practical because CVS-CPB is a common surgical procedure. Also, it is an elective procedure performed in a controlled environment where protocols can be more easily managed.

## 2 Design and Development of BMP Mimetics

### 2.1 Peptide Mimetic Design Process

**FIGURE 1:**
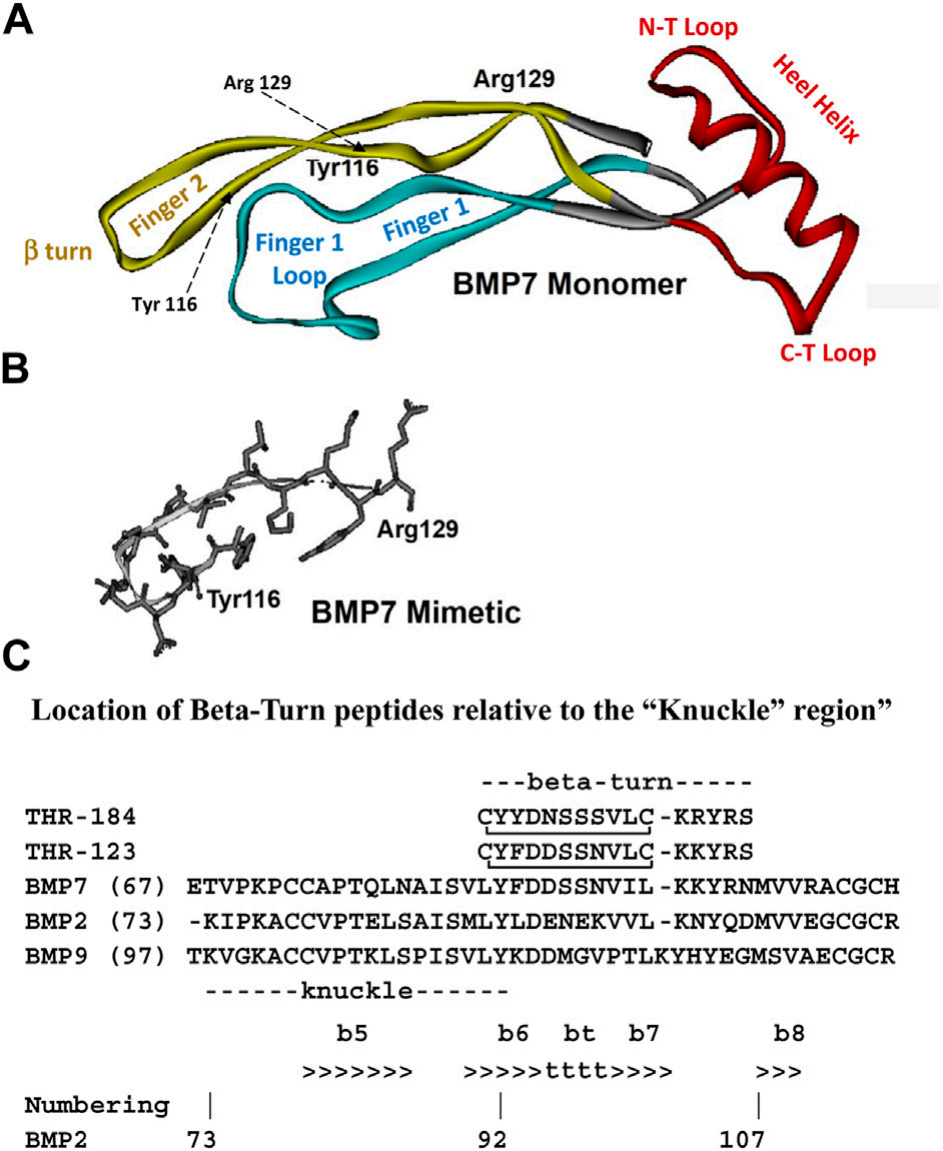
Structure diagrams of the BMP-7 monomer and the region covered by the mimetic. **(A)** A ribbon diagram showing the secondary structure of the BMP monomer, which contains three structural regions: antiparallel beta sheets of “Finger 1” (with the large terminal loop), “Finger 2” (with the tight beta-turn), and the “Heel” alpha helix. Initial targets for mimetic development were the terminal loops of fingers 1 and 2, loops at the C-terminal, and N-terminal loops at the ends of the Heel helix. **(B)** The region around the beta turn of Finger two proved to have activity similar to BMP-7 and became the lead for further mimetic development. **(C)** The “beta-turn” region covered by the mimetic is immediately C-terminal to the “knuckle” region covered by other BMP mimetics. Residue position numbers are based on BMP-2 residue numbers. Secondary structure: beta sheet (>>>>), segments of which are labelled e.g., “b6”; beta turn (bt, tttt). Peptide disulfide bond: C_C.

The starting point for the peptide mimetics considered herein were structural analogs of small regions of BMP-7 that we identified as likely receptor binding sites. The first criterion is that they have to be accessible for receptor binding. Once the three-dimensional structure of BMP-7 had been determined, the solvent-accessible regions were identified using the “rolling water probe” method of Lee and Richards (Lee and Richards, 1971). It should be noted that inherent in this identification process was the assumption that the crystal structure of BMP-7, as determined by x-ray crystallography (Griffith et al., 1996), is similar to its structure when interacting with its receptor extra-cellular domains. The second criterion is specificity. As noted above, BMP-7 is a member of a protein family that displays significant sequence homology at certain positions, which permits a clear alignment of the member sequences and implies structural homology across the family. The fact that members of the BMP family have different activities suggests that those regions displaying sequence variability across the family could also include receptor binding sites. Therefore, we selected regions of overlap between regions of putative solvent accessibility and variability as initial mimetic targets. These regions included the large loop at the end of the “finger one” anti-parallel beta strand, the tight beta turn at the end of the “finger two” anti-parallel beta strand, and the loops at the C-terminal and N-terminal ends of the “Heel” helix (Figure 1). The peptide analogs of these regions of interest were designed to include disulfide bonds intended to stabilize the conformation found in the native protein.

From this point forward the process for selection and optimization of these mimetics was based not on any assumed structural theory of receptor binding, but via their observed activity using biologically relevant *in vitro* activity and receptor binding assays. Once one or more lead peptides were identified, the same assays are used to optimize the sequence for desired activities. Binding assays measured the ability of the peptides to compete with BMP-7 in binding to cloned extracellular domains of ALKs 2, 3 and 6 as well as of BMPR-II. For cell-based *in vitro* assays, the HK2 immortalized human kidney cell line was used to look at the proinflammatory response with and without peptides of interest compared to BMP-7. *In vitro* assays were also used to look at intracellular signaling and actions such as the ability of candidate peptides, relative to that of BMP-7, to stimulate the phosphorylation of Smad-1,5,8, to block apoptosis, and to control the cell cycle. Of particular interest was the relative ability of the peptides to block the epithelial-to-mesenchymal transition (EMT) which is induced by TGF-beta signaling and is key to fibrosis development.

The peptide optimization process employed a peptide model in which each residue position is represented by a binary vector wherein each bit represents the presence 1) or absence (-1) of a physical-chemical feature such as charge, polarity, size, secondary structure propensity, etc. Based on the results from the above assays each candidate sequence is classified as either active 1) or inactive (-1). Each bit value in each sequence is multiplied by the activity classification value to create a preference value for each bit in each sequence. A statistical method, such as Bayesian analysis (Gelman et al., 1995) was employed at each bit position to create a weight, such as the log of the odds ratio of preference values, reflecting the degree to which the presence or absence of the corresponding feature favors or hinders activity. The result is a structure/activity profile based on physical/chemical features at each residue position in the mimetic, rather than on amino acid residues *per se*. This profile was then used to score the peptide sequences and to predict other likely active sequences. The optimization process proceeded as cycles of synthesizing new candidates, assaying them, and using the new data to update the structure/activity profile, etc. Once leads had been optimized, they were further evaluated in *iv vivo* animal models for kidney injury such as the Unilateral Clamp Model for ischemia/reperfusion injury. This is the optimization process used to derive THR-184 from early leads, such as THR-123.

### 2.2 BMPs as Promising Therapeutics

#### 2.2.1 TGFβ/BMP Pathways

One important area for mimetic development is the TGFβ/BMP pathways (serine/threonine kinase receptor pathways). In addition to having important and ancient roles in all stages of development, these pathways have significant roles in the adult. The BMP pathways maintain homeostasis of epithelial and endothelial cells/tissues (Wang R. N. et al., 2014), while the TGFβ pathways cause epithelial/endothelial to mesenchymal transition (EMT/EndoMT) (Zeisberg et al., 2007; Xu et al., 2009). The EMT program induces tissue fibrosis, a pathological process characteristic of most chronic diseases. The TGFβ and BMP pathways have a complementary relationship and often counterbalance each other. BMP serves as a natural antagonist of TGF beta actions (Fuchshofer et al., 2007).

The TGF beta/BMP pathways are mediated by their specific Type I and Type II receptors. Upon ligand binding, two type I and two type II receptors form an oligomeric receptor complex (Nohno et al., 1995). BMP binds any of three type I receptors: BMPR-IA (ALK-3), BMPR-IB (ALK-6) and a type IA activin receptor ActR-IA (ALK-2) (Koenig et al., 1994; Macias-Silva et al., 1998; ten Dijke et al., 1994). There are also three type II receptors involved in BMP signaling: BMPR-II and activin type II receptors ActR-IIA and ActR-IIB (Miyazono et al., 2005; Rosenzweig et al., 1995; ten Dijke et al., 1994) while TGF β interacts with the type I receptor (ALK5) and type II receptor (TGFR-II). BMP intracellular signaling is initiated when the ligand-receptor complex (Nohno et al., 1995) phosphorylates the intracellular Smads 1/5/8 (Hoodless et al., 1996; Chen et al., 1997; Nishimura et al., 1998; Tamaki et al., 1998). The phosphorylated Smad proteins then associate with the co-Smad, Smad4, and the ternary complex translocates into the nucleus where it participates in gene transcription with other transcription factors (Lopez-Rovira et al., 2002; Shi and Massague, 2003). While both type I and type II BMP receptors are required for signal transduction, specific receptors are expressed differentially in various tissues to regulate their downstream effects. Also, available evidence suggests that ALK3 preferentially mediates homeostasis of epithelial and endothelial cells (Sugimoto et al., 2012) while ALK-6 appears to mediate the osteogenic activities (Singhatanadgit and Olsen, 2011).

The selective activation of these pathways relies on the selective binding and activation of these receptors. Selective activation can thus target the desired effects and eliminate the unwanted effects.

### 2.3 BMP Activity

BMPs play important roles in regulating numerous physiological processes including cell proliferation, differentiation, apoptosis, and specification of developmental fate during embryogenesis and in adult tissues (Dudley et al., 1995; Luo et al., 1995). Aberrations in the BMP signaling pathways are associated with the pathophysiology of several diseases, including osteoporosis, arthritis, pulmonary hypertension, idiopathic pulmonary fibrosis, myocardial injury, cardiomyopathies, acute and chronic renal disease, hepatic injury, cerebrovascular diseases, and cancer.

Genetic deletion of BMP-7 in mice leads to severe impairment of kidney development, resulting in perinatal death (Dudley et al., 1995; Luo et al., 1995). Genetic defects in BMPs have also been shown to cause malformations in the heart and lung (Wang L. P. et al., 2014). The kidney is the major site of BMP-7 synthesis during embryogenesis and in the adult (Ozkaynak et al., 1991). Synthesis is confined to distal tubules, collecting ducts and podocytes of glomeruli (Vukicevic et al., 1998). BMP-7 expression decreases in several kidney disease models, including acute ischemic renal injury, tubulointerstitial fibrosis, diabetic nephropathy, and the remnant kidney model (Simon et al., 1999; Wang et al., 2001; Hruska, 2002; Dube et al., 2004). The cellular targets for BMP-7 in the kidney are convoluted tubule epithelium, glomeruli, and collecting ducts (Bosukonda et al., 2000).

In the adult, the primary biological role of BMP-7 is to maintain epithelial/endothelial cell homeostasis. BMP-7 also inhibits the induction of inflammatory cytokine expression (Sutton and Molitoris, 1998; Vukicevic et al., 1998; Gould et al., 2002), attenuates inflammatory cell infiltration, and reduces apoptosis of tubular epithelial cells in renal disease models. Thus, BMP-7 plays critical roles in repairing renal tissues. Damage to these renal tubular tissues results in kidney diseases. Accordingly, BMPs would seem to constitute a family with powerful therapeutic potential for many acute and chronic diseases, were it not for their osteoinductive activity. Since the latter activity appears to be mediated through the ALK6 type I receptor, an ideal therapeutic agent should have no or minimal affinity for the ALK6 type I receptor. The ideal BMP-7 mimetic for treating soft tissue damage necessarily would incorporate the positive properties of BMP-7 without osteoinductive activity. Moreover, these mimetics would be unlikely to interact with the natural binding protein inhibitors of the BMP’s such as follistatin, noggin, gremlin, and chordin, thus avoiding inactivation.

### 2.4 BMP Mimetics

#### 2.4.1 Related Work

There have been a number of publications describing efforts to design and test BMP mimetics (see Table 1). Most of these efforts have found compounds that are osteoinductive and therefore have limited applications to the treatment of diseases affecting soft tissues such as the heart, the lung, the liver the kidney, the pancreas, or cancers (Suzuki et al., 2000; Kirkwood et al., 2003; Saito et al., 2003). One compound has been found that is effective in cell-based assays indicating it could be used to treat pulmonary hypertension (Tong et al., 2019). The compound described is not a true mimetic since it cannot act alone, but rather enhances the effects of BMP-9. Other efforts to harness the power of the BMP pathways by using high throughput screening to find small molecules that activate the BMP pathways have had limited success (Vrijens et al., 2013; Feng et al., 2016; Bradford et al., 2019; Genthe et al., 2019).

**TABLE 1:** Other BMP agonists/mimetics.

BMP Agonist/BMP Mimetic	Derived/Designed from Type of BMP/Region	Target outcome	References
Peptide agonists	Knuckle area of BMP-2, BMP-7 and BMP-9	Induced Osteogenic activity	Suzuki et al. (2000); Kirkwood et al. (2003); Saito et al. (2003); Chen and Webster (2009)
Peptide agonist	BMP-9 knuckle area	Induced differentiation of murine preosteoblasts (MC3T3-E1 cells) and cholinergic differentiation in human SH-SY5Y neuroblastoma cells	Bergeron et al. (2009); Lauzon et al. (2017)
Peptide BFP-1/2/3	Prodomain of BMP-7	Induced stronger alkaline phosphatase activity in multipotent bone marrow stromal cells (MBSCs)	Kim et al. (2012); Kim et al. (2017); Lee et al. (2018)
Peptide	BMP-2 knuckle region	Bone inducing activity *in vivo* but did not the induce the side effects observed with BMP-2	Bain et al. (2015)
BMP mimetic peptide, P3	Wrist area of BMP-9	Enhanced BMP-9-induced Smad1/5 phosphorylation selectively in human pulmonary artery endothelial cells (hPAECs) but inhibited BMP-4-induced Smad1/5 phosphorylation in human dermal microvascular endothelial cells (HMEC-1)	Tong et al. (2019)
A cyclized BMP-7 derived peptide, THR-123	Covers the beta turn that is C-terminal to the “knuckle” of BMP-7 (see Figure 1C)	Reversed established kidney fibrosis in mouse models of chronic renal injury	Sugimoto et al. (2012)
A cyclized BMP-7 derived peptide, THR-123	Covers the beta turn that is C-terminal to the “knuckle” of BMP-7 (see Figure 1C)	Induced nongenetic conversion of human pancreatic exocrine cells to insulin-expressing and Functional (glucose-responsive) endocrine cells with a capacity for rapid reversal of diabetes *in vivo*	Qadir et al. (2020)
A cyclized BMP-7 derived peptide, THR-123, THR-184	Covers the beta turn that is C-terminal to the “knuckle” of BMP-7 (see Figure 1C)	Function as agonists of BMPR1A (BMP type I receptor), attenuated overexpression of remodeling-related genes and alleviated LV dysfunction in aortic stenosis	Salido-Medina et al. (2022)

In recent years, studies have advanced our knowledge of the cellular and systemic functions of BMPs, and therapeutics derived from BMP have been developed for the treatment of various diseases including cardio-vascular and kidney diseases (Kang et al., 2004; Rosen, 2006; Sugimoto et al., 2012; Long et al., 2015). The identification of BMP mimetics or BMP agonists remains an attractive strategy (see Table 1) due to the high cost and difficulty in manufacturing large quantities of clinical grade BMPs. Peptides that are designed from the knuckle area of BMP-2, BMP-7 and BMP-9 have been found to display similar osteogenic activity to the corresponding BMP molecule (Suzuki et al., 2000; Kirkwood et al., 2003; Saito et al., 2003; Chen and Webster, 2009). Peptides designed based on the BMP-9 knuckle area have been shown to induce differentiation of murine preosteoblasts (MC3T3-E1 cells) and cholinergic differentiation in human SH-SY5Y neuroblastoma cells (Bergeron et al., 2009; Lauzon et al., 2017). Furthermore, peptides from the prodomain of BMP-7, namely BFP-1/2/3, have also been shown to induce stronger alkaline phosphatase activity in multipotent bone marrow stromal cells (MBSCs) (Kim et al., 2012; Kim et al., 2017; Lee et al., 2018). In addition, a BMP-2 knuckle peptide has been shown to possess similar bone inducing activity *in vivo* but did not induce the side effects observed with BMP-2 (Bain et al., 2015).

BMP peptides hold the potential for improving specificity. Tong et al. (Tong et al., 2019) identified a BMP-9 mimetic peptide P3, designed from the “wrist area” of BMP-9, which enhanced BMP-9-induced Smad1/5 phosphorylation selectively in human pulmonary artery endothelial cells (hPAECs) but inhibited BMP-4-induced Smad1/5 phosphorylation in human dermal microvascular endothelial cells (HMEC-1). Although these findings are exciting, very few studies have performed in-depth binding and mechanistic investigation of how these peptides achieve their specificity.

A cyclized BMP-7 derived peptide, THR-123, a member of the BMP-7 mimetic family, which covers the beta turn that is C-terminal to the “knuckle” (see Figure 1C), was reported to successfully reverse established kidney fibrosis in mouse models of chronic renal injury (Sugimoto et al., 2012).

More recently it has been shown that THR123, effectively induced nongenetic conversion of human pancreatic exocrine cells to insulin-expressing and Functional (glucose-responsive) endocrine cells with a capacity for rapid reversal of diabetes *in vivo* (Qadir et al., 2020). This work independently demonstrates a safer and simpler alternative to genetic reprogramming. Also, THR123 and another BMP-7 mimetic, THR184, function as agonists of BMPR1A (BMP type I receptor), attenuated overexpression of remodeling-related genes (*Col 1α1*, *β-MHC*, *BNP*), palliated structural damage (hypertrophy and fibrosis) and alleviated left ventricular (LV) dysfunction in a thoracic aortic constriction animal model (Salido-Medina et al., 2022).

### 2.5 THR-184

A mimetic peptide of BMP-7 was designed and optimized using the process outlined above. The sequences of the approximately 30 members of the human BMP subfamily of the TGF-beta superfamily have several strictly conserved residues throughout the sequences, which suggests a high degree of structural homology across the subgroup. The x-ray structure of BMP-7, which retains a high degree of three-dimensional structural homology with other superfamily members (Griffith et al., 1996), was used to identify solvent accessible sites. The knuckle region covered by the osteogenic peptides and the beta-turn region covered by the non-osteogenic mimetic peptides described here are separate structural regions (Figure 1C). This separation allows small mimetic peptides to be designed with specific activity.

The receptor binding and *in vitro* assay activity, indicating half maximal effective concentrations (EC_50_) of the lead peptide variants, were used for structure/activity analysis to arrive at THR-184 as the prime clinical candidate**.**

### 2.6 Characterization of the Mimetic

**FIGURE 2:**
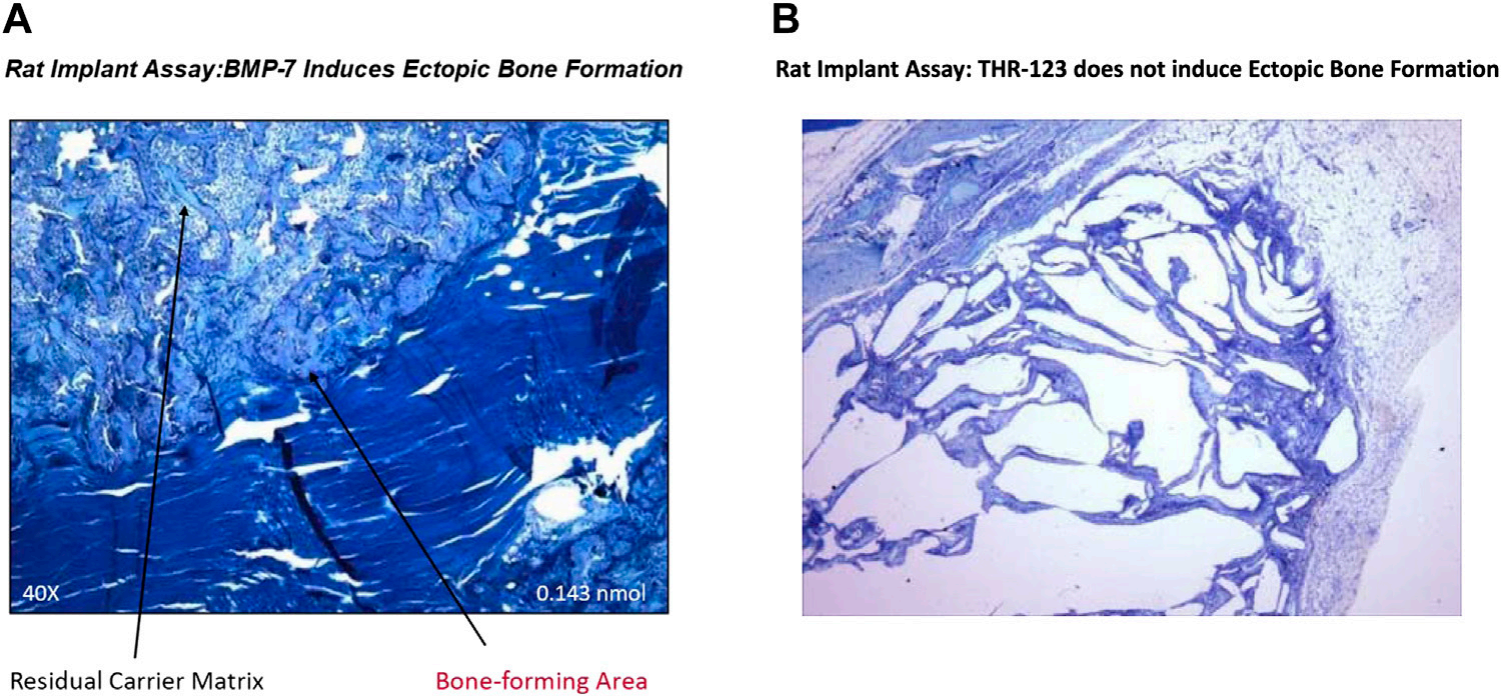
Failure of BMP-7 mimetic (THR-123) to induce ectopic bone formation in rat implant assay: This assay is based on the ability of BMP-7 to induce ectopic bone formation in the rat and was carried out as described earlier (*The Journal of Biological Chemistry*, Vol. 267, pp. 20352-20362,1992) to determine *in vivo* efficacy of THR-123. Briefly, bovine bone matrix (collagen carrier)/BMP-7 or Hydrogel/BMP-7 or Hydrogel/THR-123, devices (all lyophilized) were implanted in muscle pocket. Implants removed on day 14 for were analyzed by histology (stained by toluidine blue) and results indicated that THR-123 **(** Figure 2B) failed to induce ectopic bone formation, while BMP-7 (Figure 2A) which served as a positive control induced bone formation. This work was supported by Thrasos Therapeutics.

The THR-184 and THR-123 are members of the BMP-7 mimetic family. The family of BMP mimetics are agonists that signal through the activin-like kinase three receptor (ALK3). The BMP-7 mimetics are effective in initiating the phosphorylation of Smads 1, 5 and 8, blocking apoptosis, and have the ability to suppress renal tubule cell inflammation. Also, the mimetic (THR-123) failed to induce ectopic bone formation in a rat implant assay (Figures 2A, B). Preclinical work with THR-184 (Table 2) in multiple animal models showed suppression of inflammation, apoptosis, reversal of the epithelial-to-mesenchymal transition (EMT) program, and attenuation of renal injury, including ischemia/reperfusion injury. Moreover, there was no evidence of bone formation in Pre-clinical Tox experiments. Thus, the mimetic, unlike BMP-7, showed no osteoinduction.

**TABLE 2:** THR-184 overview.

A novel therapeutic that activates the BMP pathway and minimizes AKI
Peptide mimetic of the active portion of BMP-7
Binds BMP type I receptors ALK2 and ALK3, but not ALK6
Prevents apoptosis, cytokine release and fibrosis
Does not induce bone growth
Does not promote cancer growth
Does not inhibit wound healing
Does not affect bleeding time
Does not affect hemodynamics
Has no off-target interactions (114 targets surveyed)
Has no effect on key CYP450 isoforms (1A2, 2C19, 2C9, 2D6, 3A4)

Imaging studies of radio-labeled mimetic injected via the tail vein in rats showed distribution primarily to the kidney cortex (Sugimoto et al., 2012).

### 2.7 Target Disease Selection

#### 2.7.1 Organ of Choice: Kidney

As mentioned previously, several lines of evidence indicate that the kidney would be a good target organ for the clinical investigation of a BMP mimetic. The kidney is the major site of BMP-7 synthesis during embryogenesis and in the adult (Ozkaynak et al., 1991). Expression is confined to distal tubules, collecting ducts and podocytes of the glomeruli (Vukicevic et al., 1998). BMP-7 expression decreases in several kidney disease models, including acute ischemic renal injury, tubulointerstitial fibrosis, diabetic nephropathy, and remnant kidney models (Simon et al., 1999; Wang et al., 2001; Hruska, 2002; Dube et al., 2004). The cellular targets for BMP-7 in the kidney are the convoluted tubule epithelium, glomeruli, and collecting ducts (Bosukonda et al., 2000). In animal models proximal tubule damage can be rescued by systemic infusion of exogenous BMP-7 (Sutton and Molitoris, 1998; Vukicevic et al., 1998). Therefore, the kidney was chosen as the target organ for clinical testing and development of the mimetic. Further, for the reasons presented below, ischemic injury to the kidney was thought to be a viable clinical target.

### 2.8 Target Disease

A particularly pernicious consequence of ischemic injury is Acute Kidney Injury (AKI). AKI is defined as an abrupt (within hours) decrease in kidney function that encompasses both renal structural damage and loss of kidney function. The term AKI has largely replaced Acute Renal Failure (ARF), reflecting the recognition that smaller decrements in kidney function that do not result in overt organ failure are, nevertheless, of substantial clinical relevance and are associated with increased morbidity and mortality. Recent evidence from both basic medical science and clinical research, is beginning to change our view of AKI from a single-organ failure syndrome to a syndrome where the kidney plays an active role in the progress of multi-organ dysfunctions (Makris and Spanou, 2016).

Injury induced by ischemia can result in damage to both the tubular and the microvascular compartment. Resolution of vasoconstriction appears effective at reducing injury when administered prophylactically, but not following established injury. Resistance may be due to exacerbated inflammation, which may impart reductions in RBF^1^ and GFR insensitive to vasodilator therapies. Of central importance in this process is the activation of inflammatory processes which are influenced by factors released by damaged proximal tubules as well as adhesion of damaged microvascular cells. Infiltrating leukocytes may impinge on RBF either by secreting vasoactive factors, or by contributing to the disruption of flow by physical interference. In addition, exacerbated hypoxia leading to tubular obstruction may contribute to reductions in GFR independent of vasodilator therapy (Basile et al., 2012).

**FIGURE 3:**
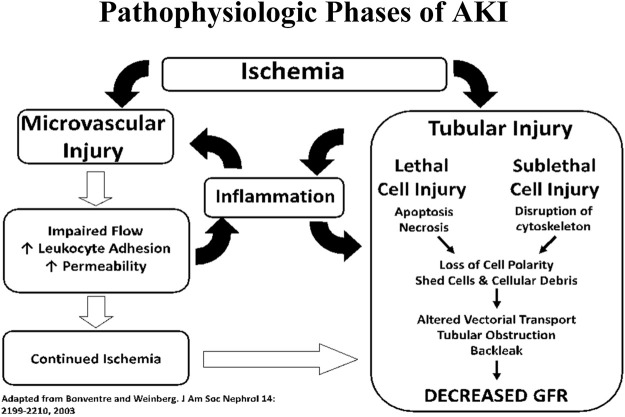
Interplay between tubular and vascular injury leading to sustained reductions of GFR in the extension phase of AKI.

Mortality in patients admitted to the hospital with AKI has been estimated to be 20% in patients not in the ICU and 50% in patients admitted to the ICU. Thus far, AKI has defied attempts to find an effective treatment, let alone a cure (Brown et al., 2010). The pathophysiology of AKI (see Figure 3) involves disruption of arterial and venous flow to the kidney as well as injury to various structures within the kidney such as the glomerulus, the collecting ducts, and tubular interstitium (Basile et al., 2012; Case et al., 2013). The primary targets of injury in AKI are the renal proximal tubule epithelial cells (RPTECs) lining the tubules proximal to the glomerulus. Damaged cells sluff-off and clog the downstream tubules and collecting ducts. This clogging lowers the effective Glomerular Filtration Rate (GFR) and affects other functional aspects of the nephron. The two primary sources of injury to RPTECs are: toxins, such as heavy metals, and ischemia/reperfusion (ischemia and the subsequent immune response to reperfusion).

AKI tends to occur in the sickest patients, often requiring the most complicated treatments. These patients are typically undergoing major surgery and/or in need of critical care (Askenazi et al., 2006; Lo et al., 2009; Brown et al., 2010; Mammen et al., 2012). Current therapies are predominantly supportive. They endeavor to promote urine output, to obtain adequate renal perfusion, to prevent fluid overload, to reduce the buildup of toxic metabolites, and to optimize hemodynamics and promote renal blood flow (Askenazi et al., 2006; Mammen et al., 2012). The intensive investigation of AKI reflects the fact that none of the standard therapeutic strategies employed to treat AKI are able to regulate the multiple pathways involved in AKI pathophysiology. Consequently, to date, there are no established pharmacotherapies for AKI.

The complexities of AKI constitute the kind of pharmacotherapeutic challenge that suggests a more robust approach, such as the use of growth factors, might be more effective. The results of several such trials are mixed. Exogenous IGF-1, which is beneficial in the recovery after kidney injury in mouse models, failed to demonstrate the efficacy in patients with acute renal failure (ARF) (Hirschberg et al., 1999). A double-blind placebo-controlled trial, designed to test whether early treatment with erythropoietin could prevent the development of AKI in ICU patients (Endre et al., 2010), failed to show a difference between the placebo and treatment groups. Currently, a small hepatocyte growth factor/scatter factor mimetic, ANG-3777, that has been shown to improve renal function in patients after kidney transplantation (FDA, 2015) is undergoing a clinical trial in patients who are susceptible to kidney injury (Hulse and Rosner, 2019). This limited success with treatments for AKI strengthens our above-described interest in a mimetic of the BMP pathway.

A successful AKI therapeutic would also likely lead to major improvements in quality of life during aging and to major annual health budget savings. Recent studies have demonstrated a relationship between AKI and the subsequent development of chronic renal insufficiency, a progression to hemodialysis and other severe consequences (Askenazi et al., 2006; Coca et al., 2009; Ishani et al., 2011; Mammen et al., 2012). Even mild renal injury can have long term deleterious effects (James et al., 2020).

Thus, there is a large body of evidence indicating that AKI can lead to chronic kidney disease CKD. AKI is thought to be driven by inflammation and apoptosis. The long-term effects of these processes lead to CKD and are known to involve fibrosis.

### 2.9 Diagnosis

The current standards for the diagnosis of AKI rely on the loss of functional markers, such as serum creatinine-based estimates of GFR, but such functional markers do not accurately reflect the degree of actual renal injury. Ultimately, an AKI incident that escapes detection could lead to the development of CKD for which hemodialysis is a maintenance treatment, but not a cure.

#### 2.9.1 Clinical Model

The incidence of cardio-vascular disease requiring surgical treatment is high and often leads to the development of perioperative AKI. The incidence of AKI during and after cardio-vascular surgery in which a cardiopulmonary bypass pump (CVS-CPB) is used (Englberger et al., 2011; Bastin et al., 2013; James et al., 2014; Machado et al., 2014; Quan et al., 2016), or that is associated with vascular surgery (Kashani et al., 2015; Quan et al., 2016; Warren et al., 2016) is as high as 20–70% of cases, depending on the type of surgery and the definition of AKI used for diagnosis. Furthermore, a large percentage of patients who receive complex Cardio-vascular Surgery (CVS) are elderly with multiple comorbidities that predispose them to the development of AKI and potentially hasten progression to End Stage Renal Disease (ESRD) (Lysak et al., 2020). In recent years, there have been considerable advances in our understanding of CVS-associated AKI (CVS-AKI), but, despite the high prevalence, there is little consensus about how best to prevent or treat CVS-AKI.

iData Research estimated that there were 340,000 Coronary Artery Bypass Graft Surgery (CABG) procedures done in the US in 2017, and over 900,000 cardiac surgeries performed each year prior to 2018. Approximately 84.2% of these surgeries were performed with the use of cardiopulmonary bypass (iData Research, 2021). CABG procedures done using cardiopulmonary bypass with a cardioplegic solution when the heart’s pumping action is arrested, and the patient’s circulatory system is temporarily maintained using a cardiopulmonary bypass machine. The mortality after open heart surgery without AKI is between 1 and 8% (Karkouti et al., 2009), but increases more than fourfold in patients with AKI (Karkouti et al., 2009). In patients who require Renal Replacement Therapy (RRT) post operatively, the mortality is as high 63% (Thakar et al., 2005; Vives et al., 2019).

The choice of CVS-AKI for the Phase two clinical trial was determined by the sizeable patient population having CVS-CPB surgery and an unmet need for a way to control the morbidity and mortality due to perioperative AKI. The primary factor of concern with this model, however, is that serious preoperative comorbidity such as CKD and diabetes, while adding to the incidence of AKI, also renders the trial results more difficult to interpret.

#### 2.9.2 Clinical Outcome Endpoints

The primary functional marker for AKI is the glomerular filtration rate (GFR). While a drop in GFR is a clear sign of nephrological problems, a normal GFR is no guarantee of kidney health. This is because the kidney has excess clearance capacity that masks underlying renal injury. It has been estimated that an individual needs only 10% of their normal kidney filtration capacity to have adequate function.

Added to the fundamental limitations of GFR as a measure of injury, the primary surrogate marker for estimating GFR (eGFR) is the level of creatinine in blood serum (sCr) and urine. sCr is a GFR marker that is a product of muscle secreted creatinine in blood serum, which is removed by clearance through the kidney nephron. A steady state is achieved when the rate of creatinine production equals the rate of creatinine clearance. A rise in sCr is, therefore, the combined result of a decrease in the rate of renal clearance and an increase in the rate of Cr production due to metabolic abnormalities or high muscle mass. Determining the degree to which Cr production is a factor is difficult. Consequently, sCr is a noisy surrogate marker of kidney function, let alone kidney injury.

Furthermore, 20–40% of the creatinine found in urine from a normally functioning kidney, avoids filtration through the glomerulus by being secreted by the renal tubules and reabsorbed by the distal tubules. This process, referred to as creatinine excretion, becomes more prevalent when there is injury to the proximal tubules. It masks issues such as blockage in the nephron’s Loop of Henley due to cells sluffed off because of injury. Thus, Cr-based eGFR is a poor marker for renal injury in that it can overestimate the true GFR.

#### 2.9.3 Previous Therapeutic Strategies

There have been many different attempts to prevent AKI after CVS that target single pathways. Observational studies using calcium channel blockers (Antonucci et al., 1996), (Bergman et al., 1995), (Colson et al., 1992) failed to protect against AKI during thoracic aortic cross-clamping (Pass et al., 1988; Lassnigg et al., 2000). Likewise atrial natriuretic peptide in AKI also failed to show beneficial renoprotective effects (Rahman et al., 1994). Although Angiotensin Converting Enzyme inhibitor (ACEi) or Angiotensin Receptor Blocker (ARB) therapy should be avoided in most cases, the use of intravenous enalaprilat (Wagner et al., 2003) has improved kidney performance in patients who have undergone coronary artery bypass complicated by left ventricular dysfunction. Intravenous pentoxifylline in elderly patients could be beneficial, but more studies are needed to assess its efficacy (Boldt et al., 2001). In a randomized, single-blind, controlled pilot trial of 120 adult patients undergoing cardiopulmonary bypass, remote ischemic preconditioning resulted in a 27% absolute risk reduction of AKI (Zimmerman et al., 2011).

Thus, intensive investigations of AKI reflect the fact that none of these therapeutic strategies could regulate the multiple pathways involved in inflammation, fibrosis, mitochondrial function, apoptosis, oxidative stress, and hemodynamics while simultaneously promoting regeneration. Details have been well described in recent reviews (Nadim et al., 2018; Vives et al., 2019; Gameiro et al., 2020; Ostermann et al., 2021). There is now effort to address multiple pathways with a growth factor mimetic. A small molecule hepatocyte growth factor/scatter factor (HGF/SF) mimetic, termed ANG-3777, is undergoing clinical trial in patients who are susceptible to kidney injury (Hulse and Rosner, 2019). Investigators from Angion Biomedica Corp have demonstrated that ANG-3777 improves renal function in patients after kidney transplantation (FDA, 2015). Furthermore, research by this company is assessing whether ANG-3777 can reduce the severity of delayed graft function in recipients of a deceased donor kidney. A phase two study to assess the safety and efficacy of ANG-3777 in patients who develop AKI after cardiac surgery is ongoing (Ayad et al., 2020).

### 2.10 Route of Administration and Dose Range Selection

A reliable ischemia-reperfusion animal model for testing the protective and therapeutic power of BMP mimetics proved to be the Unilateral Clamp Ischemic Reperfusion rat model developed by Bruce Molitoris (Sutton and Molitoris, 1998). This model was used to determine the clinical-trial dosing of the BMP mimetic THR-184. In this model, animals undergo unilateral nephrectomy and clamping of the renal artery of remaining kidney. A 30-min renal artery occlusion was used to produce ischemia reperfusion and trigger apoptosis and minimize necrosis. The model was used to assess efficacy and to establish dose ranges for different modes of delivery. The two modes of administration tested were IV bolus and IV infusion of up to 1 h, before and after clamping.

In this rat model THR-184 produced a dose-dependent reduction in serum creatinine levels with an ED_50_ of ∼10–30 µg/kg, with maximal efficacy (65% reduction in serum creatinine levels) measured with a 1 mg/kg dose. That is a ∼100-fold ED_50_ shift toward greater potency from the bolus to the infusion.

**FIGURE 4:**
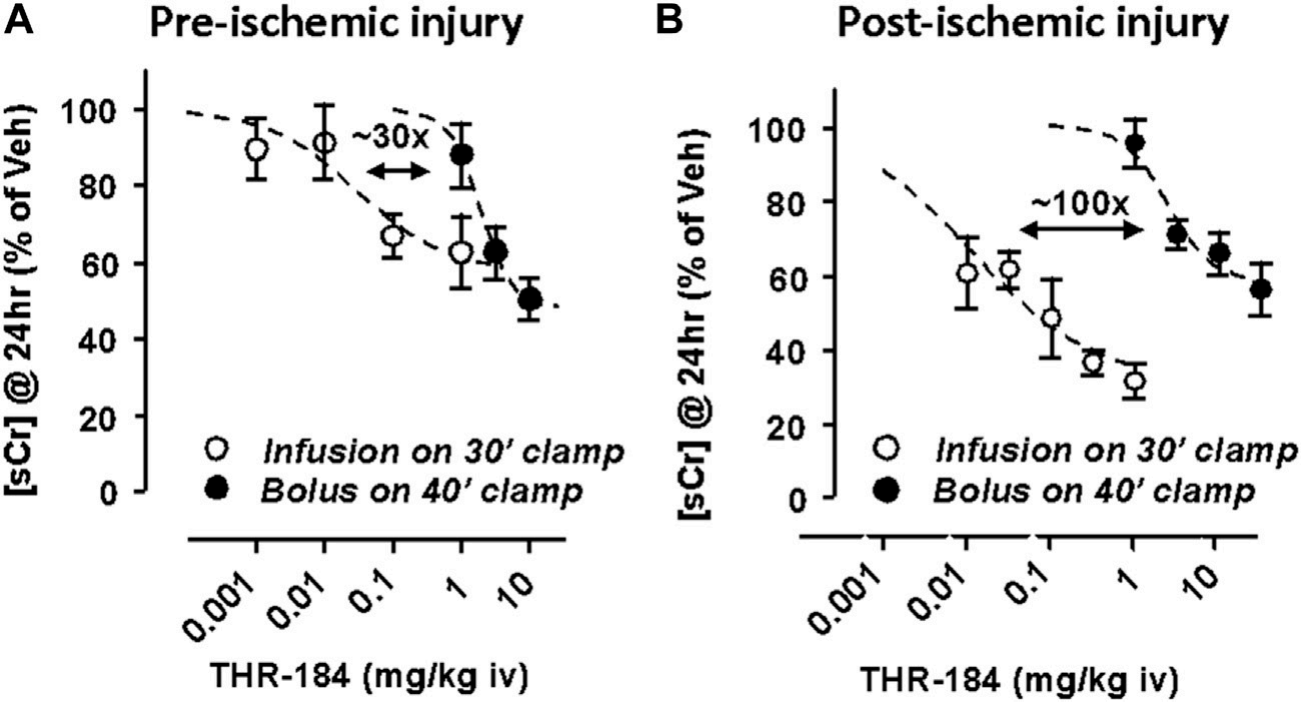
Unilateral Clamp Ischemic Reperfusion model in the Rat^2^. This work was supported by Thrasos Inoovations.

Similarly, a 1 h infusion administered prior to the ischemic insult produced a dose-dependent reduction in serum creatinine levels with an ED_50_ ∼10 μg/kg. Longer infusion times (4 and 24 h) provided no additional efficacy compared to the 1 h infusion. In a pre-ischemic injury model, the same reduction in sCr at 24 h was obtained via slow infusion using ∼30x less THR-184 than was required via bolus injection (Figure 4A). In the post-ischemic injury mode, the same reduction in sCr at 24 h was obtained via slow infusion using ∼100x less THR-184 than was required via bolus injection (Figure 4B).

One kidney is removed and the blood supply to the other is clamped off for 30–40 min. Figure 4A: The effect of treatment pre-ischemic insult using a slow infusion of THR-184 versus that of a bolus injection. The same reduction in [sCr] at 24 h was obtained via slow infusion using ∼30x less THR-184 than was required via bolus injection. Figure 4B: The effect of treatment post-ischemic insult using a slow infusion THR-184 versus that of a bolus injection. The same reduction in [sCr] at 24 h was obtained via slow infusion using ∼100x less THR-184 than was required via bolus injection. In addition, the maximum degree of reduction is greater using a slow infusion than via a bolus injection.

The 1-h post-ischemic infusion was found to require one percent of the total dose of the IV bolus to produce the same reduction in the rise of serum creatinine. Thus, the doses for the clinical trial were scaled down conservatively and it was decided that an IV infusion of 1 hour would be administered 1 hour prior to initiation of cardiopulmonary bypass and an IV infusion of 1 hour would be administered daily for 3 days post operatively.

### 2.11 Phase 1 Clinical Trials - Safety in Healthy Human Volunteers

Two phase 1 safety studies, each a randomized, adaptive, double-blind, placebo-controlled study at a single center, evaluated safety, tolerability, and pharmacokinetic behavior of ascending doses of THR-184. These were done at Algorithme Pharma of Laval, Quebec, Canada, under FDA Guidelines, and their study numbers for single Ascending Dose Trial and Multiple Ascending Dose Trial were THR-P1-427 and THR-P1-428, respectively.

#### 2.11.1 Single Ascending Dose (SAD) Trial

In the first study, THR-P-427 (SAD), the drug (THR-184) was administered as slow intravenous infusions in a total of 40 adult healthy male and female volunteers. The study drug or placebo was administered once on the morning after a 10-h overnight fast. The solution was administered by slow injection via an indwelling catheter, at an infusion rate of 1 ml/min over 60 min. Five dose levels were studied: 0.3 mg/kg, 1 mg/kg, 3 mg/kg, 6 mg/kg and 10 mg/kg. Adverse events were recorded continuously during the study. There were no serious adverse events observed and the most frequently observed adverse event was headache which occurred equally in the placebo and THR-184 treated subjects. No significant adverse events or deaths were reported during this study. No adverse events required the use of medications. No significant abnormal laboratory values, vital signs, ECG’s, physical examinations, or neurological examinations were observed in any subject.

#### 2.11.2 Multiple Ascending Dose (MAD) Trial

In the second study, THR-P-428 (MAD), THR-184 was administered as a slow intravenous infusion in a total of 24 adult healthy male and female volunteers, once daily over five consecutive days. THR-184 or the placebo was administered on the morning after a 10-h overnight fast. The solution was administered by slow injection via an indwelling catheter, at an infusion rate of 1 ml/min over 60 min. Three dose levels were studied: 0.3 mg/kg, 1 mg/kg, and 3 mg/kg. Adverse events were recorded continuously during the study. There were no serious adverse events observed and the most frequently observed adverse event was somnolence which occurred equally in the placebo and THR-184 treated subjects. No significant adverse events or deaths were reported during this study. No adverse events required the use of medications. No significant abnormal laboratory values, vital signs, ECG’s, physical examinations, or neurological examinations were observed in any subject.

In summary, this Phase 1 Trial of THR-184, as described in “Perioperative THR-184 and AKI after Cardiac Surgery” (Himmelfarb et al., 2018), found no toxicity or adverse events in healthy human volunteers.

#### 2.11.3 Phase II Clinical Trial

The objective of the Phase II clinical trial, NCT01830920, (Thrasos Innovation, Inc, 2013), was to establish the dose, safety, and preliminary efficacy of THR-184 in a targeted patient population having major cardio-vascular surgery requiring the use of the cardiopulmonary pump The primary outcome of this clinical trial was the proportion of patients who develop AKI according to the KDIGO criteria (Kidney Disease: Improving Global Outcomes (KDIGO) Acute Kidney Injury Work Group, 2012). The KDIGO criteria were on the basis of a positive result for any of three measures: 1) increase in serum creatinine by ≥ 0.3 mg/dl (≥26.5 μmol/L) within any 48 h 7 days after surgery, 2) increase in serum creatinine to ≥1.5 times baseline within 7 days after surgery, or 3) urine volume <0.5 ml/kg per hour for six consecutive hours. Use of urinary creatinine is not part of the KDIGO AKI criteria, but rather it is the urinary output that is part of the AKI definition. Because of the difficulty of defining a clinically efficacious dosing range for an agonist therapeutic, the trial employed an adaptive design where several dose levels were tested in the first half of the trial and, following a third-party analysis of these data, two doses were selected to be continued through the second half of the study. The clinical trial included serum samples for the analysis of KIM-1 and NGAL as exploratory endpoints, but there are as yet no published results from those samples.

#### 2.11.4 Trial Results

Table 3 lists the results for the full dataset of the trial. The Phase II patient population was diverse. It included individuals ranging from those with mild AKI to those entering the trial with significant pre-existing chronic kidney disease (CKD). This turned out to be a significant and informative factor in the trial outcome. The measure of outcome accepted by the FDA is function, which for AKI means sCr levels and amounts in urine as surrogate measures of GFR. As mentioned above, because most individuals are born with excess renal capacity, GFR is minimally affected by the loss of function until the capacity is down to about 20%. Thus, in individuals with relatively healthy kidneys, GFR grossly underestimates the level of AKI until the disease has become critical.

**TABLE 3:** Dose definitions (One Pre/Three Post surgery): Arm 2 - 0.02-mg/kg/0.02-mg/kg; Arm 3 - 0.12-mg/kg/0.02-mg/kg; Arm 4 - 0.46-mg/kg/0.02-mg/kg; Arm 5 - 0.46-mg/kg/0.46-mg/kg. KDIGO criteria for diagnosing AKI: Increase in SCr by ≥ 0.3 mg/dl (≥26.5 μmol/L) within 48 h OR Increase in SCr to ≥1.5 times baseline, which is known or presumed to have occurred within the prior 7 days OR Urine volume <0.5 ml/kg/h for 6 h. KDIGO Cr: The full KDIGO criteria minus the Urine volume criterion. sCr >0.3: Increase in sCr by ≥ 0.3 mg/dl (≥26.5 μmol/L) within 48 h sCr >50%: Increase in serum creatinine ‡50% within 7 days. SAE: number of Serious Adverse Events. *p*-value: Unadjusted *p* value (two sided) from logistic regression with baseline eGFR as a covariate, comparing the active arm with arm 1 (placebo) (Himmelfarb et al., 2018).

Analysis of phase 2						
Arm	1	2	3	4	5	
	Placebo	Low Dose	Mid Dose	High Dose	Highest Dose	
**Subjects**	**113**	**34**	**34**	**116**	**104**	**#**
**KDIGO**	**77.9**	**79.4**	**76.5**	**75.9**	**74.0**	**%**
***p*** **-value**		**0.76**	**0.95**	**0.76**	**0.59**	
**sCr > 0.3**	**53.1**	**58.8**	**55.9**	**55.2**	**51.9**	**%**
**sCr > 50%**	**20.4**	**32.4**	**23.5**	**20.7**	**22.1**	**%**
**Urine Output**	**60.2**	**58.8**	**55.9**	**51.7**	**61.5**	**ml/kg/hr**
**KDIGO Cr**	**54.9**	**58.8**	**58.8**	**56.0**	**51.9**	**%**
**SAE > 1**	**41.7**	**46.7**	**59.6**	**42.0**	**41.0**	**%**
**Deaths**	**3**	**4**	**5**	**4**	**5**	**#**

There are several confounding factors that could influence the protective effects of the BMP mimetic in AKI. These factors include the compound’s pharmacokinetic and pharmacodynamic properties. In the clinical trial, the pharmacokinetic data show that there is a clear relationship of the dose to serum levels of the compound. However, variability in the serum levels of the compound was large, producing a large standard deviation in both maximum serum concentration (C Max) and Arear Under the Curve (AUC). The magnitude of this variability is on the order of the measured serum levels themselves, which confound any measured beneficial effects in the kidney. As mentioned previously, in animal studies, it has been observed that changing from a bolus injection to an infusion decreased the ED_50_ by a factor of 30 when the compound THR-184 was administered prior to renal injury and a factor of 100 when it was administered immediately after the injury (Figures 3A, B). The serum half-life of THR-184 was determined to be 5 minutes, which increased to 60 min when compound was administered with an ACE inhibitor.

Three factors - 1) the primary effect on tubular secretion, 2) short half-life of the compound in plasma and 3) the large variability of C Max and AUC - are certainly confounding factors that make it difficult to see the beneficial effect measured by serum creatinine in patients with AKI. A longer infusion or co-administration with an ACE inhibitor could dramatically increase the beneficial effect in the clinical trial. Taken together, these results suggest that changes to the protocol with a higher sample size per cohort could produce a statistically significant effect of THR 184 on the incidence of AKI in patients undergoing cardio-vascular surgery.

We believe that our data is best understood when analyzed by a valid alternative method: comparison between the groups can be based on the proportion of group members (%) yields a more accurate view of the clinical trial outcome because of the inherent high variability of creatinine values observed, and the small sample size in each cohort. In a recent phase two study (NCT01286727) to evaluate the efficacy and safety of HGF/SF mimetic (ANG-3777) for improving kidney function in patients at high risk for delayed graft function after kidney transplantation (Bromberg et al., 2021), Bromberg observed a similar discrepancy in results, which they attributed to low participant sample size. Consequently, they began a Phase 3 study based on the effect size rather than statistical significance (Angion Biomedica Corp, 2021).

#### 2.11.5 Subgroup Analysis

There were 40 patients (out of 104) that entered the high dose arm of the trial with pre-existing CKD (Thrasos Innovation, Inc, 2013; Himmelfarb et al., 2018). Despite the small number of patients, as shown in Table 4, analysis of this subgroup showed some provocative results. The CKD patients, eGFR <60 ml/min/1.73 m^2^, responded well when treated with THR-184 as indicated by an 11–17% reduction in AKI. Patients in this subgroup that also received an ACE inhibitor post-surgery had an even greater reduction (25 vs. 34%) (Carlson, 2018). In the patients that completed the study without protocol violations and were classified as having Stage 1 AKI, there was a 27% decrease in those treated with the highest dose compared to those in the placebo group. This result suggests the dosing for this trial may only have been at the beginning of the therapeutic window (Thrasos Innovation, Inc, 2013). There were no safety concerns.

**TABLE 4:** +Data drawn from Table S1 Himmelfarb et al. (2018) * Data drawn from Table S4 Himmelfarb et al. (2018).

Subgroup Analysis of Phase 2							
Arm	1 placebo	2 Low Dose	3 Mid Dose	4 High Dose	5 Highest Dose	%Diff (1-5)	
**Subjects After Surgery**	**113**	**34**	**34**	**116**	**104**		**#**
**KDIGO**	**77.9**	**79.4**	**76.5**	**75.9**	**74.0**	**3.9**	**%**
***p*** **-value**		**0.76**	**0.95**	**0.76**	**0.59**		
**sCr KDIGO**	**54.9**	**58.8**	**58.8**	**56.0**	**51.9**	**3.0**	**%**
**sCr>0.3 mg/dl and**	**53.1**	**58.8**	**55.9**	**55.2**	**51.9**	**1.2**	**%**
**Per Protocol**							
**Subjects Completed +**		**24**	**29**	**91**	**71**		**#**
**KDIGO +**	**78.5**	**75.0**	**75.9**	**78.0**	**70.4**	**8.1**	**%**
**sCr KDIGO +**	**57.0**	**54.2**	**58.6**	**56.0**	**49.3**	**7.7**	**%**
**sCr >0.3mg/dl +**	**55.9**	**54.2**	**55.2**	**54.9**	**49.3**	**6.6**	**%**
**Per Protocol: eGFR < 60 ml/min/1.73m^2^**							
Subjects Completed *	**48**	**--**	**--**	**--**	**31**		**#**
KDIGO *	**85.4**	**--**	**--**	**--**	**74.2**	**11.2**	**%**
sCr KDIGO *	**75.0**	**--**	**--**	**--**	**58.1**	**16.9**	**%**
Stage 1 AKI *	**62.5**				**35.5**	**27.0**	**%**

However, the results based on the KDIGO endpoint did not show a statistically significant difference between the AKI and placebo groups. It has been pointed out that when the outcome variability is high and the sample size is small, a valid alternate way to compare the groups can be based on the proportion of group members being classified in an outcome category. This is a better way than relying on level of significance for comparison of the groups (Schober et al., 2018).

It is noteworthy that the compound showed reduced incidence of AKI in the population with pre-existing chronic kidney disease. The reduction of AKI was even more dramatic in those patients with pre-existing chronic kidney disease that received concomitant treatment with an ACE inhibitor (Carlson, 2018).

Dose definitions (One Pre/Three Post surgery): Arm 2 - 0.02-mg/kg/0.02-mg/kg; Arm 3 - 0.12-mg/kg/o. o2-mg/kg; Arm 4 - 0.46-mg/kg/0.02-mg/kg; Arm 5 - 0.46-mg/kg/0.46-mg/kg. KDIGO criteria for diagnosing AKI: Increase in SCr by ≥ 0.3 mg/dl (≥26.5 μmol/L) within 48 h OR Increase in SCr to ≥1.5 times baseline, which is known or presumed to have occurred within the prior 7 days OR Urine volume <0.5 ml/kg/h for 6 h. KDIGO Cr: The full KDIGO criteria minus the Urine volume criterion. sCr >0.3: Increase in sCr by ≥ 0.3 mg/dl (≥26.5 μmol/L) within 48 h sCr >50%: Increase in serum creatinine >50% within 7 days. SAE: number of Serious Adverse Events. Stage 1 AKI: sCr 1.5 - 1.9 times baseline OR sCr≥0.3 mg/dl (≥26.5 μmol/L) increase, Urine output <0.5 ml/kg/hr for 6–12 h.

## 3 Discussion

### 3.1 THR-184 Treatment Effectiveness

For the reasons outlined above, AKI is a difficult disease to treat. Clinical trials are confounded by several complexities which are hard to control in a randomized clinical trial. Cardio-vascular surgery has many confounding variables that can affect the occurrence of AKI. These include time on cardiopulmonary bypass, hypotension, anesthetic agents, pressors, inotropes, diuretic use, intravascular volume shifts, and blood loss. Currently, there is no pharmacological intervention that has consistently been associated with renal protection (see Section 2.9.3). This failure is likely related to the following:

• AKI after cardiac surgery is pathophysiologically multifactored and complex, thus simple strategies targeting a single pathophysiological factor and/or single pathway will most likely fail.
• End points based on GFR, and especially sCr-based eGFR, will show little effect except in those patients entering the trial with low renal capacity.
• eGFR is a non-linear and low sensitivity indicator of underlying acute renal injury that, left untreated, can lead to loss of renal capacity and lead to the development of CKD.
• When the clinical outcome variability is high, and renal injury varies from mild to severe, the patient pool participating in the dosed arm vs. placebo should be sufficiently large.
• Finally, most clinical data come from trials conducted on low-risk patient populations that have a lower prevalence of AKI.

Biomarkers such as KIM1 and NGAL, although not yet approved as clinical surrogate endpoints, have the advantage of being early markers of renal injury because their concentration rises within a few hours after injury (Mishra et al., 2005; Han et al., 2008; Khreba et al., 2019). The available evidence from our preclinical studies with THR-184 supports the use of these biomarkers in an AKI clinical trial. In animal models, THR-184 provided nephroprotection from Ischemic Reperfusion Injury and Cisplatin-induced AKI by preventing renal tubular damage, inhibiting tubular apoptosis, and decreasing urinary NGAL (Carlson, 2018). We included KIM-1, NGAL and Albumin as exploratory endpoints in our Clinical trial (NCT01830920). Accordingly, serum and urine samples were obtained pre-, during, and post-treatment with THR184. However, they remain to be assayed. Our understanding is: Sample analysis for these markers is not part of either the primary or secondary outcomes of the clinical trial. However, the availability of data from sample analysis for the KIM-1 and NGAL markers will likely add to our understanding of THR-184 efficacy and the use of these markers in AKI. Importantly, previous studies have found that, as early markers, KIM-1 and NGAL can help to diagnose AKI with high sensitivity and specificity (Bonventre, 2008; Devarajan, 2011; Geng et al., 2021).

AKI is increasingly recognized as a major risk factor for progression to CKD after cardiac surgery (Hsu and Hsu, 2016; Mizuguchi et al., 2018). Multiple signaling pathways including SMAD (Vigolo et al., 2019), p38 MAPK (Terada et al., 2007) and Wnt/β-catenin (Xiao et al., 2016) are implicated as mediators of the progression of AKI into CKD. We have observed that the BMP mimetic alone can regulate these pathways. Importantly, the BMP mimetic has a protective role in the AKI-CKD transition by regulating the WNT/β-catenin signaling pathway. Also, it is capable of inhibiting renal inflammation and blocking/reversing epithelial-mesenchymal transition (EMT), one of the hallmarks of the AKI-CKD transition. In our clinical trial, the effect of THR-184 was more prominent in AKI patients with limited renal capacity due to underlying CKD.

Patient population definition is critical in situations where functional endpoint markers can be insensitive to the targeted clinical condition. Most clinical data come from trials conducted on low-risk patient populations. In the case of this trial, where the functional marker, eGFR, is sensitive to the underlying injury only after there is a significant loss of renal capacity, clinical significance is much easier to achieve when the patient pool is limited to those with pre-existing low renal capacity.

Clinical trial designs are problematic where the injury condition does not affect functional markers in the near term but, if left untreated, does have ultimate long-term implications for function. Trials having long term endpoints require long term follow-up, but the per-protocol patient population decays with time due to the inevitable follow-up protocol violations. This kind of long-term trial will be very large, long, and expensive.

### 3.2 Perioperative THR-184 Phase two Clinical Trial

In the report “Perioperative THR-184 and AKI after Cardiac Surgery” on the results of the Phase two clinical trial (Himmelfarb et al., 2018), the trial’s renal advisory board and the chief scientific officer of our company, Thrasos Innovation, state on page 670 that they “found that administration of perioperative THR-184 through a range of dose exposures failed to reduce the incidence, severity, or duration of AKI in patients with high-risk cardiac surgery” (Himmelfarb et al., 2018).

There were two important findings in this publication. The first concerns the safety of the compound, THR-184. THR-184 at all doses tested was well tolerated by the patients and was shown to have no adverse effects in treated patients compared to the Placebo. The second finding concerns the efficacy of THR-184 on AKI after cardiac surgery in high-risk patients. The study pointed out that “a higher postoperative THR-184 dose arm showed lower incidence of post-surgery AKI relative to placebo; the difference, although not statistically different, was more pronounced among patients with preexisting CKD in the per protocol dataset” (Himmelfarb et al., 2018). This is certainly a significant advance since there have been no effective therapeutics to reduce AKI after cardiac surgery.

While the phase two trial did not show statistically significant effects, it did provide data that points to the next steps for clinical development. We understand Phase two Clinical Trials as defined by the Food and Drug Administration:

“In Phase two studies, researchers administer the drug to a group of patients with the disease or condition for which the drug is being developed. Typically involving a few hundred patients, these studies are not large enough to show whether the drug will be beneficial.

“Instead, Phase two studies provide researchers with additional safety data. Researchers use these data to refine research questions, develop research methods, and design new Phase 3 research protocols” (https://www.fda.gov/patients/drug-development-process/step-3-clinical-research).

In the significance statement on p. 671 Himmelfarb et al. note that “The lack of observed clinical benefit (in the trial) is similar to that in other recently completed randomized clinical trials” (Himmelfarb et al., 2018). This, we contend, will be the result for any clinical trial of a therapeutic that addresses renal injury so long as the end point is based solely on functional markers. Failure is a loss of function, which is the result of injury, but there can be injury without detectable loss of function. Therapeutics such as THR-184 treat underlying renal injury. The intent is to prevent loss of function, but as noted above, there is mounting evidence that multiple episodes of even minor AKI ultimately lead to Chronic Kidney Disease (Askenazi et al., 2006; Coca et al., 2009; Ishani et al., 2011; Mammen et al., 2012; James et al., 2020). Unfortunately, the current clinical definition of AKI involves functional parameters such as estimated Glomerular Filtration Rate (eGFR), which are poor surrogate markers of underlying renal injury when there is excess renal capacity. Only in the case of patients entering the trial with reduced renal capacity, where they are closer to the point where loss of function is detectable, will functional markers be reliable in diagnosing actual injury (see Table 3). In this context, we took a closer look at the trail results for the subpopulation of patients entering the trial with preexisting CKD (Table 4 above).

In Table 4, the incidence of AKI, as judged by the sCr KDIGO standard, was 57.0% in the placebo arm and 49.3% in the highest dose arm. This is a reduction of 7.7%. In the Per Protocol subgroup analysis of patients with eGFR <60 the incidence of AKI, as judged by the sCr KDIGO standard, was 75.0% in the placebo arm and 58.1% in the highest dose arm. This is a 16.9% reduction. The incidence of Stage 1 AKI in the Per Protocol population was 47.0% for the placebo arm and 29.0% for the highest dose arm. This is an 18% reduction. In the Per Protocol subgroup analysis of patients with eGFR <60 the incidence of AKI, as judged by the sCr KDIGO standard, was 62.5% in the placebo arm and 35.5% in the highest dose arm. This is a 27.0% reduction. Thus, while not statistically significant due to the small sample size of this subpopulation and the masking of the injury due to the use of eGFR, there is evidence that THR-184 reduced acute renal injury.

Because of the afore-mentioned correlation between incidences of AKI and ultimate development of CKD (Askenazi et al., 2006; Coca et al., 2009; Ishani et al., 2011; Mammen et al., 2012; James et al., 2020), it is important to monitor the level of renal injury during surgical episodes. One way to assess the degree of renal injury independent of functional markers is by monitoring specific renal injury markers such as KIM-1 and NGAL. In fact, the design for the Phase two trial included the collection of samples for assaying the levels of KIM-1, NGAL and Albumin (see Himmelfarb Table 2). Unfortunately, there has been no published report of those data.

### 3.3 Considerations in New Trials

#### 3.3.1 Biomarkers–Injury vs Function

Because AKI can exist without detection via functional markers in patients that have little or no pre-surgery CKD, markers of cellular injury are important in an AKI study for two reasons. First, AKI events contribute the eventual development of CKD. Second, AKI that is not detected by functional markers still puts stress on the remaining nephrons, which can lead to a spreading of the AKI. Functional detection of this spiraling injury may come too late in the process to be halted therapeutically leading to patient death.

#### 3.3.2 CKD

Given the excess capacity issue with using functional markers of GFR to diagnose AKI, discussed above, these biomarkers could be more sensitive to the condition in a patient population having pre-existing CKD.

#### 3.3.3 Clinical Endpoints

Given the significance of undiagnosed AKI, injury markers such as KIM-1 and NGAL could improve the clinical outcome of AKI trials. The correlation between therapeutic treatment and the levels and duration of injury markers will likely give a better assessment of mimetic efficacy, even when functional markers are negative.

#### 3.3.4 Bioavailability

**FIGURE 5:**
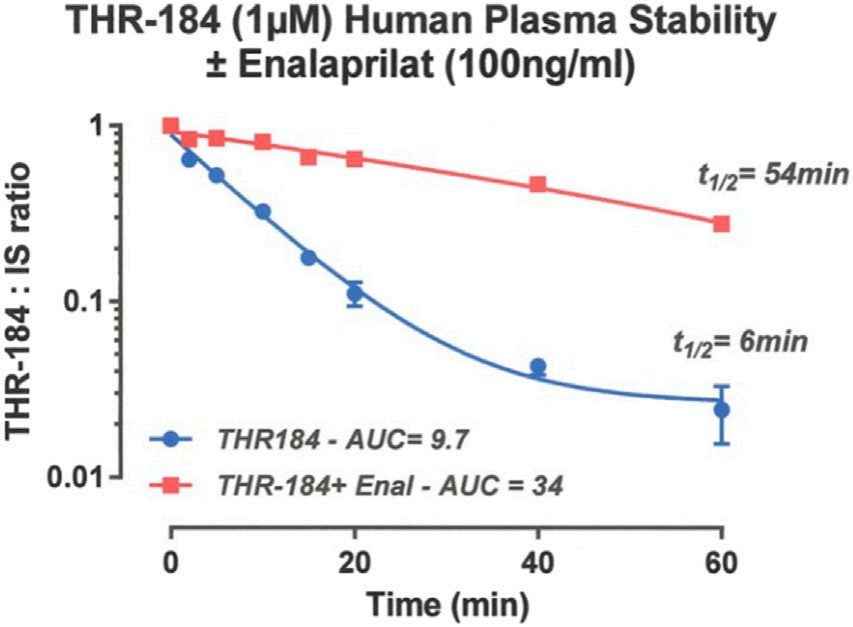
Pharmacokinetics of THR-184: Stability of THR-184 in Human plasma in the presence and absence of ACE inhibitor (Carlson, 2020).

Protease degradation of drug candidates can shorten their in-plasma half-life to the point that therapeutic exposure is severely compromised resulting in loss of efficacy. We have observed that the serum half-life of THR 184 was 5 minutes, which increased to 60 min when the compound was administered with an ACE inhibitor, suggesting that an ACEi could improve the bioavailability of the BMP mimetic. This result (Figure 5), taken with the pharmacokinetic data, suggests that an infusion of up to 24 h would potentially be more effective.

#### 3.3.5 ACEi

The beneficial pleiotropic effects of ACE inhibitors go far beyond blood pressure reduction (Joannidis and Hoste, 2018). It is noteworthy that in the above study the small number of CKD patients that were put on the BMP mimetic with an ACEi faired noticeably better than those given the mimetic without an ACEi.

## 4 Summary and Conclusion

Growth factors and cytokines regulate complex signaling networks. Through their multiple interactions they initiate repair and alter the growth, differentiation, and metabolism of target cells. As therapeutics, growth factors have presented challenges because they are proteins with pleiotropic effects, are difficult and expensive to manufacture, and frequently require parenteral administration. We have undertaken an effort to develop compounds that mimic the beneficial effects of the naturally occurring ligands/growth factors while having more drug-like properties and the potential to be orally administered. We have chosen to focus on the BMP family of growth factors. There are at least 30 different, naturally occurring members in the human BMP family. All of them possess structural homology and diverse activities. They act through an array of specific receptors that stimulate multiple signaling pathways. They regulate the downstream pathways to effect homeostasis and the repair of specific tissues and organs. Newly developed BMP mimetics can use the pleiotropic effects of the BMPs to advantage by selectively activating the BMP receptors. These properties make the BMP mimetic system a useful model for the development of other growth factor mimetics. The BMP mimetics discussed here are novel peptide agonists of the BMP signaling pathways. They are relatively small and are designed using *in vitro* and *in vivo* assay data and algorithms that identify structure-activity relationships. They are optimized to selectively possess beneficial effects and eliminate the unwanted deleterious effects of naturally occurring full-length BMP. They are simple and economical to synthesize.

The primary biological role of BMP-7 is to maintain epithelial/endothelial cell homeostasis. As a proof of concept, we designed a family of BMP mimetics that binds specifically to the kidney receptors. These mimetics initiate a signaling cascade that decreases renal epithelial inflammation and apoptosis, reduces tubular damage, and augments renal regeneration. These effects produce an attenuation of renal dysfunction. One member of the family of BMP-7 mimetics, THR-184, has progressed through Phase 1 and Phase 2 clinical trials. The goal of the phase two proof-of-concept study was to provide guidance for future work by assessing the dosing, safety, and efficacy of THR-184 in patients at high risk of AKI induced by cardiac surgery involving cardiopulmonary bypass. The compound was shown to be safe and well tolerated. The results of this trial provide a roadmap for future trials to develop treatments for renal injury.

The results based on a KDIGO endpoint did not show a statistically significant difference between THR-184 treated and placebo groups of AKI, but there was a noticeable reduction in the incidence of AKI in the patient subgroup with pre-existing CKD treated with the highest dose of THR-184. The results also showed an even higher reduction in AKI patients with mild chronic renal insufficiency who were also treated with an angiotensin-converting enzyme inhibitor (ACE inhibitor). This suggests that patients with chronic renal insufficiency would be a better choice for a clinical trial.

There are other markers to consider, such as Kim-1 and NGAL, that are more sensitive markers for the detection of renal injury. The use of these in AKI clinical trials could provide better diagnosis of renal injury and help forecast the long-term effects.

We believe that the development of growth factor mimetics, and in particular the BMP-7 mimetics, offers great promise for the treatment of acute kidney injury and other serious debilitating diseases. This novel approach may point the way toward the development of therapeutics that capture the potential inherent in growth factors. The ability of the BMP-7 mimetics to block apoptosis, the inflammatory response to injury, reverse fibrosis and regulate differentiation in soft tissues could provide therapeutics with broad applications to the treatment of acute and chronic diseases. The potential for regulating cellular differentiation and reversing EMT could open the door for therapeutics that regenerate the damaged tissue in failing organs.

## 5 Significance statement

Naturally occurring growth factors are critical agents that control a myriad of biological processes. The design of growth factor mimetics holds the potential for the development of therapeutics that could repair and regenerate injured tissue and control cancer. Growth factors themselves have several barriers to their use as therapeutics. This Hypothesis/Theory articulates methods by which the power of the biological processes controlled by growth factors could be harnessed to develop therapeutics with drug-like properties. For example, the process of developing BMP mimetics can produce compounds that incorporate the beneficial properties and eliminate the deleterious effects of the naturally occurring growth factors. This process of designing small peptide mimetics with drug like properties activating the BMP-7 signaling pathways provides a roadmap for the design and the clinical development of many therapeutics. These mimetics could treat, and potentially cure, a myriad of diseases by repairing and regenerating injured tissues. The magnitude of this impact would be momentous and represents a new paradigm in drug development.

## Data Availability

The original contributions presented in the study are included in the article/Supplementary Material, further inquiries can be directed to the corresponding author.

## Footnotes

1 Renal Blood Flow (RBF).

2 Sutton, T. A., and Molitoris, B. A (1998). Mechanisms of cellular injury in ischemic acute renal failure. Semin Nephrol, 18(5), 490-497. https://www.ncbi.nlm.nih.gov/pubmed/9754601
